# A phase Ib study of utomilumab (PF-05082566) in combination with mogamulizumab in patients with advanced solid tumors

**DOI:** 10.1186/s40425-019-0815-6

**Published:** 2019-12-04

**Authors:** Ezra E. W. Cohen, Michael J. Pishvaian, Dale R. Shepard, Ding Wang, Jared Weiss, Melissa L. Johnson, Christine H. Chung, Ying Chen, Bo Huang, Craig B. Davis, Francesca Toffalorio, Aron Thall, Steven F. Powell

**Affiliations:** 1 UC San Diego Health, Moores Cancer Center, University of California San Diego, 3855 Health Sciences Drive, La Jolla, CA 92093 USA; 2 Georgetown University Medical Center, Washington, DC USA; 3 Cleveland Clinic, Cleveland, OH USA; 4 Henry Ford Hospital, Detroit, MI USA; 5 University of North Carolina at Chapel Hill, Chapel Hill, NC USA; 6 Sarah Cannon Research Institute, Nashville, TN USA; 7 Moffit Cancer Center, Tampa, FL USA; 8 Pfizer Inc, La Jolla, CA USA; 9 Pfizer Inc, New York, NY USA; 10 Pfizer Inc, Milan, Italy; 11 Sanford Research, Sioux Falls, SD USA

**Keywords:** 4-1BB, CD137, Utomilumab, Mogamulizumab, Solid tumors

## Abstract

**Background:**

Expressed on activated T and natural killer cells, 4-1BB/CD137 is a costimulatory receptor that signals a series of events resulting in cytokine secretion and enhanced effector function. Targeting 4-1BB/CD137 with agonist antibodies has been associated with tumor reduction and antitumor immunity. C-C chemokine receptor 4 (CCR4) is highly expressed in various solid tumor indications and associated with poor prognosis. This phase Ib, open-label study in patients with advanced solid tumors assessed the safety, efficacy, pharmacokinetics, and pharmacodynamics of utomilumab (PF-05082566), a human monoclonal antibody (mAb) agonist of the T-cell costimulatory receptor 4-1BB/CD137, in combination with mogamulizumab, a humanized mAb targeting CCR4 reported to deplete subsets of regulatory T cells (Tregs).

**Methods:**

Utomilumab 1.2–5 mg/kg or 100 mg flat dose every 4 weeks plus mogamulizumab 1 mg/kg (weekly in Cycle 1 followed by biweekly in Cycles ≥2) was administered intravenously to 24 adults with solid tumors. Blood was collected pre- and post-dose for assessment of drug pharmacokinetics, immunogenicity, and pharmacodynamic markers. Baseline tumor biopsies from a subset of patients were also analyzed for the presence of programmed cell death-ligand 1 (PD-L1), CD8, FoxP3, and 4-1BB/CD137. Radiologic tumor assessments were conducted at baseline and on treatment every 8 weeks.

**Results:**

No dose-limiting toxicities occurred and the maximum tolerated dose was determined to be at least 2.4 mg/kg per the time-to-event continual reassessment method. No serious adverse events related to either treatment were observed; anemia was the only grade 3 non-serious adverse event related to both treatments. Utomilumab systemic exposure appeared to increase with dose. One patient with PD-L1–refractory squamous lung cancer achieved a best overall response of partial response and 9 patients had a best overall response of stable disease. No patients achieved complete response. Objective response rate was 4.2% (95% confidence interval: 0.1–21.1%) per RECIST 1.1. Depletion of Tregs in peripheral blood was accompanied by evidence of T-cell expansion as assessed by T-cell receptor sequence analysis.

**Conclusions:**

The combination of utomilumab/mogamulizumab was safe and tolerable, and may be suitable for evaluation in settings where CCR4-expressing Tregs are suppressing anticancer immunity.

**Trial registration:**

ClinicalTrials.gov identifier: NCT02444793.

## Background

Cancer immunotherapy, in particular monoclonal antibody (mAb) antagonists of the programmed cell death protein 1 (PD-1)/programmed cell death-ligand 1 (PD-L1) pathway, has substantially helped patients with a variety of solid tumor types, including non–small-cell lung cancer (NSCLC) [1], squamous cell carcinoma of the head and neck (SCCHN) [2], melanoma [3], bladder cancer [4], and renal cell carcinoma [5]. Patients whose tumors do not respond to PD-1/PD-L1 antagonists represent an increasingly recognized area of unmet need [6]. Tumor cell extrinsic mechanisms, such as the lack of T cells or the presence of immunosuppression [7], may define a subclass of patients who would benefit from combinations that provide costimulatory signals to antitumor T cells while removing immunosuppressive cells. One such combination is utomilumab plus mogamulizumab.

Utomilumab (PF-05082566) is a fully human immunoglobulin G2 agonist mAb that binds to human 4-1BB/CD137 with high affinity and specificity [8]. 4-1BB/CD137 is a costimulatory receptor of the tumor necrosis factor receptor superfamily expressed on activated immune cells, including T cells [9], dendritic cells [10], and natural killer cells [11]. 4-1BB/CD137 agonists promote immune cell proliferation, survival, cytokine production [12–15], formation of immunologic memory, and sustained T-cell immune responses [16–18]. Lymphocyte activation and favorable antitumor responses have been elicited by utomilumab as well as other 4-1BB/CD137 agonists in multiple preclinical models [8, 13, 19–21]. A phase I trial of utomilumab recently reported a favorable safety profile and preliminary antitumor activity [22].

Mogamulizumab is a recombinant humanized mAb targeting C-C chemokine receptor 4 (CCR4, CD194). It was first approved in Japan in 2012 for relapsed or refractory CCR4^+^ adult T-cell leukemia-lymphoma (ATL), and approval for first-line treatment of CCR4^+^ ATL was granted in 2014. Approval for additional indications of relapsed or refractory CCR4^+^ peripheral T-cell lymphoma and cutaneous T-cell lymphoma was gained in 2014. In 2018 it was approved by the US Food and Drug Administration and European Medicines Agency for the treatment of relapsed or refractory mycosis fungoides or Sézary syndrome after at least 1 prior systemic therapy. CCR4 has been observed on regulatory T cells (Tregs) [23]. In vitro or in vivo mogamulizumab treatment selectively depleted CCR4^+^ Tregs and is associated with increased levels of tumor-antigen–specific T cells [24, 25].

The combination hypothesis has been evaluated by in vivo experiments in a murine melanoma model in which the antitumor activity of 4-1BB/CD137 was significantly improved when given in combination with an anti-CD4 mAb that depleted Tregs as well as other CD4^+^ cells [26]. Anti–4-1BB/CD137 treatment resulted in the polyclonal expansion and differentiation of antitumor CD8^+^ T cells into effective antitumor agents, whereas CD4^+^ T-cell depletion facilitated the infiltration of immune cells into the tumors and removed Treg hindrance [26].

The mechanistic data for utomilumab and mogamulizumab as single agents coupled with the preclinical outcomes supported clinical evaluation of the hypothesis that depletion of CCR4^+^ Tregs by mogamulizumab would enhance the efficacy of antitumor immune responses expanded by utomilumab.

This phase Ib study investigated safety, efficacy, pharmacokinetics (PK), and pharmacodynamics of utomilumab plus mogamulizumab in patients with advanced solid tumors previously unresponsive to currently available therapies or for whom no standard therapy was available.

## Patients and methods

### Study design and objectives

This phase I, open-label, multicenter, multiple-dose study was approved by the institutional review boards at all nine centers in the US. Patients were enrolled between May 26, 2015 and February 7, 2017 (study completion October 10, 2017). The study was conducted in compliance with the ethical principles originating in or derived from the Declaration of Helsinki and in compliance with the International Council for Harmonization Good Clinical Practice Guidelines. All patients provided written informed consent. The study is registered on ClinicalTrials.gov (NCT02444793).

The primary objective of the study was to estimate the maximum tolerated dose (MTD) of utomilumab in combination with mogamulizumab in patients with advanced solid tumors. Secondary objectives included assessment of the safety profile, PK, immunogenicity, and antitumor activity of the combination. Exploratory objectives included the pharmacodynamic effect on immune parameters in blood.

### Patients

Refractory patients had a previously documented best overall response (BOR) of non-complete response (CR)/partial response (PR)/stable disease (SD) on PD-1/PD-L1 treatment (includes progressive disease and clinical deterioration); relapsed patients had documented BOR of CR/PR/SD but later progressed on PD-1/PD-L1 treatment (includes progressive disease and clinical deterioration).

Patients were not eligible if they had a history of autoimmune disease; systemic anticancer therapy within 28 days prior to registration; radiation therapy within 14 days prior to registration; therapeutic or experimental mAbs within 28 days prior to registration; active and clinically significant bacterial, fungal, or viral infection; live vaccine within 30 days prior to registration; or systemic corticosteroid therapy or any other form of immunosuppressive therapy within 14 days prior to registration.

### Treatment

The starting dose for intravenous utomilumab was 1.2 mg/kg every 4 weeks, with escalation to 2.4 mg/kg and 5 mg/kg in the subsequent cohorts following the time-to-event continual reassessment method (TITE-CRM). A flat dosing of 100 mg utomilumab was also assessed. Intravenous mogamulizumab 1 mg/kg was administered weekly for 4 consecutive weeks and biweekly thereafter, following utomilumab dosing. Treatment with study drugs was to continue until the first occurrence of one of the following: completion of 24 months of treatment, disease progression, patient refusal to continue, unacceptable toxicity, or study termination by the sponsor.

### Study assessments

#### Safety

Safety assessments included dose-limiting toxicities (DLTs) in the first 2 cycles and adverse events (AEs) characterized by type, frequency, severity (as graded by National Cancer Institute Common Terminology Criteria for Adverse Events version 4.03). Causality was first assigned by the site Principal Investigator and then all serious AEs (SAEs) were adjudicated at a regular conference involving all sites and sponsor. The following AEs were considered DLTs if they were attributable to one or both study drugs: grade 4 neutropenia, febrile neutropenia, grade ≥ 3 neutropenic infection, grade ≥ 3 thrombocytopenia with bleeding, grade 4 thrombocytopenia, grade ≥ 3 non-hematologic abnormalities, and grade 4 aminotransferase/alanine aminotransferase increase. The MTD was defined as the highest combination dose with a DLT rate < 30% from the TITE-CRM model estimate.

#### PK and immunogenicity

Blood for utomilumab PK assessment was collected at predose and end of utomilumab infusion on Day 1 of Cycles 1–4; on Day 1 at predose and end of utomilumab infusion; at 2, 6, and 168 h (Day 8) and 336 h (Day 15) after the start of utomilumab infusion in Cycle 5; predose on Day 1 of Cycles 8, 12, 16, 20, and 24; and end of treatment (EOT). PK samples for mogamulizumab were collected at predose and end of mogamulizumab infusion on Days 1, 8, 15, and 22 of Cycle 1; predose of Cycles 2–4; on Day 1 at predose and at the end of mogamulizumab infusion, and at 6 and 168 h after the start of the mogamulizumab infusion, and predose on Day 15 of Cycle 5; predose on Day 1 of Cycles 8, 12, 16, 20, and 24; and EOT. Samples were assayed using validated enzyme-linked immunosorbent assays in compliance with standard operating procedures of the study sponsor (Pfizer, New York, NY, USA) for utomilumab and of Kyowa Hakko Kirin (KHK; Tokyo, Japan) for mogamulizumab. Standard serum PK parameters were estimated for both drugs using non-compartmental analysis.

Blood samples for antidrug antibody (ADA) assessments were collected at predose on Day 1 of Cycles 1, 3, 5, 8, 12, 16, 20, and 24, and EOT. If ADAs were detected, additional samples were collected approximately every 8 weeks until ADA levels returned to baseline. Serum samples were assayed for ADAs using a validated electrochemiluminescence (ECL) assay (anti-utomilumab) and ECL-based ligand-binding assay (anti-mogamulizumab) in compliance with standard operating procedures of the sponsor (anti-utomilumab) and KHK (anti-mogamulizumab). ADA-positive samples were further tested for neutralizing antibodies (NAb) using a validated cell-based luciferase assay (anti-utomilumab) or ECL-based ligand-binding assay (anti-mogamulizumab).

#### Pharmacodynamic assessments

Readouts included changes in peripheral blood biomarkers, including cytokines, distribution of lymphocyte subpopulations, and frequency of T-cell receptor (TCR) sequences. Blood was collected for immunomodulation/cytokine-release biomarkers at pre-infusion of utomilumab on Day 1 and at the end of mogamulizumab infusion for Cycles 1–4; pre-infusion of utomilumab on Day 1, at the end of utomilumab infusion, and 2 and 6 h after the start of the utomilumab infusion of Cycle 5. Blood for the characterization of lymphocyte subpopulations was collected at predose on Day 1, and 2, 6, and 168 h (Day 8) and 336 h (Day 15) after the start of infusion of Cycles 1 and 5.

Analysis of serum cytokines and lymphocyte subpopulations in peripheral blood was performed as described by Tolcher et al. [27]. Lymphocyte subpopulations in the current report were defined using CD45, CD3, CD4, CD8, CD25, CD127, CD45RA, and CCR7. Expanded TCR sequences were quantified as described by Rytlewski et al. [28]. Statistical assessments of effects seen in the flow cytometry and TCR sequence expansion analyses were performed using Wilcoxon signed-rank testing and Wilcoxon rank-sum testing, respectively.

#### Characterization of baseline tumor biopsies

Immunohistochemistry was performed to detect the presence of PD-L1, CD8, FoxP3, and 4-1BB/CD137 in the whole tumor and invasive margin (IM) of pretreatment tumor biopsies. Immunohistochemistry testing of PD-L1 (clone E1L3N; Cell Signaling, Danvers, MA), CD8 (clone C8/144B; Dako, Carpinteria, CA), FoxP3 (clone 236A/E7; Cell Signaling), and 4-1BB/CD137 (BBK-2; ThermoFisher, Rockford, IL) was performed by Mosaic Laboratories, LLC (Lake Forest, CA).

#### Antitumor activity

Radiologic tumor assessments were conducted at baseline within 28 days prior to treatment, and on treatment every 8 weeks, starting from Cycle 1 Day 1 (up to 1 year), then every 3 months. Assessments were also to be conducted whenever disease progression was suspected, at EOT, and during follow-up visits. Response was assessed using RECIST1.1. Objective response was defined as BOR of CR or PR from the date of first dose of study treatment until disease progression. Both CR and PR were confirmed by repeat assessments performed no fewer than 4 weeks after the criteria for response were first met.

#### Statistical analyses

A modified TITE-CRM method with cyclical adaptive weight function was applied [29, 30]. The MTD was estimated as the highest dose level associated with a < 30% estimated DLT rate per the modified TITE-CRM design. A dose-escalation steering committee was established to facilitate the trial conduct process [31]. A sample size of 30 was estimated to provide an accurate estimate of the MTD and to detect any unexpected toxicity occurring at 5% rate (in a non–dose-dependent fashion), with a probability of 0.79, and occurring at 10% rate with a probability of 0.96. The objective response was summarized with objective response rate (ORR), and exact 2-sided 95% confidence interval (CI) for ORR was calculated using the Clopper–Pearson method. Time-to-event endpoints were analyzed using the Kaplan–Meier method. Point estimates of Kaplan–Meier rates and median times were presented with their 95% CIs. The CIs for the median were calculated according to the Brookmeyer and Crowley method.

## Results

### Patients and treatment

In all, 24 patients received mogamulizumab 1 mg/kg in combination with utomilumab dosed as follows: 1.2 mg/kg (*n* = 11), 2.4 mg/kg (*n* = 4), 5 mg/kg (*n* = 3), and 100-mg flat dose (*n* = 6). Most patients were male (79.2%) and white (79.2%). The mean (range) age was 63.9 (53–75) years. There were 11 patients with SCCHN, 10 with NSCLC (*n* = 7 squamous and 3 adenocarcinoma), 2 patients with colorectal cancer, and 1 patient with ovarian cancer, assessed by Response Evaluation Criteria in Solid Tumors (RECIST 1.1). The majority (91.7%) of patients had received at least 2 lines of anticancer drug therapy **(**Table 1**)**. Median (range) duration of treatment was 16 (4.0–41.3) weeks. All of the patients with squamous NSCLC (*n* = 7), 1 with lung adenocarcinoma, and 7 with SCCHN were relapsed or refractory to anti–PD-1/PD-L1 checkpoint inhibitor therapy. Nine (37.5%) and 15 (62.5%) patients had baseline Eastern Cooperative Oncology Group performance status 0 and 1, respectively. The dose-expansion phase of the study was not initiated due to marginal efficacy.

**Table 1:** Primary diagnosis and prior anti-cancer treatment

Number (%) of patients	Mogamulizumab 1 mg/kg + Utomilumab, by Dose Group				
	1.2 mg/kg n = 11	100 mg *n* = 6	2.4 mg/kg *n* = 4	5 mg/kg *n* = 3	Total *n* = 24
CRC	*1*	0	1	0	2
NSCLC	2	6	1	1	10
Ovarian Cancer	0	0	0	1	1
SCCHN	8	0	2	1	11
Prior anti-cancer drug regimens					
1	1 (9.1)	0	1 (25.0)	0	2 (8.3)
2	3 (27.3)	3 (50.0)	0	0	6 (25.0)
3	2 (18.2)	2 (33.3)	1 (25.0)	3 (100.0)	8 (33.3)
≥ 4	5 (45.5)	1 (16.7)	2 (50.0)	0	8 (33.3)

*CRC* Colorectal cancer, *NSCLC* Non-small-cell lung cancer, *SCCHN* Squamous cell cancer of head and neck

### Safety

No DLTs were observed at any utomilumab dose (1.2 mg/kg, 2.4 mg/kg, 5 mg/kg, 100 mg flat dose) in combination with mogamulizumab 1 mg/kg. Although no DLTs were observed up to 5 mg/kg, the estimated recommended Phase II dose was at least 2.4 mg/kg per the TITE-CRM method; as the 5 mg/kg cohort only enrolled 3 patients, this dose was not fully explored in this respect. The most common (in ≥25% of patients), all-causality AEs were fatigue (45.8%), rash (29.2%), and diarrhea (25.0%), all of grade 1 or grade 2 severity. Eight (33.3%) patients experienced all-causality grade 3–4 AEs. Ten (41.7%) patients experienced serious AEs (SAEs), all determined to be unrelated to utomilumab or mogamulizumab; AE causality was initially assessed by the site Principal Investigator and all SAEs were adjudicated during regular conferences involving all sites and sponsor. The majority of the treatment-related AEs were grade 1 or 2, and none were grade 4 or 5. Two (8.3%) patients in the utomilumab 100 mg/mogamulizumab 1 mg/kg treatment group experienced three grade 3 AEs determined to be related to treatment: pneumonitis (utomilumab-related), hypophosphatemia (mogamulizumab-related), and anemia (both treatments). Three (12.5%) patients experienced grade 5 AEs, determined to be unrelated to either treatment. Of these patients, 2 occurred within 30 days after the last dose of study treatment and were due to malignant neoplasm progression/disease progression. The third patient died due to sepsis during the follow-up period (within 60 days after the last dose of study treatment).

### PK and immunogenicity

Five patients had sufficient data to calculate PK parameters at Cycle 5. Utomilumab systemic exposure based on area under the serum concentration–time curve to last measureable dose and maximum serum concentration values appeared to increase with increasing dose. Due to the low number of patients, the relationship between serum PK parameters and dose could not be fully determined **(**Table 2**)**.

**Table 2:** Descriptive summary of serum utomilumab pharmacokinetic parameter values for Cycle 5

Parameter, Units	Mogamulizumab 1 mg/kg + Utomilumab, by Dose Group^a^		
	1.2 mg/kg	100 mg	5 mg/kg
*n*	2	1	2
AUC_last,_ μg·h/mL	907, 1440	2700	1620, 9270
AUC_last,_(dn), μg·h/mL/mg/kg	756, 1200	2490	323, 1850
C_max,_ μg/mL	17.5, 20.0	27.8	86.6, 129
C_max,_(dn), μg/mL/mg/kg	14.6, 16.7	25.6	17.3, 25.8
C_trough,_ μg/mL	1.30^b^	1.16	2.19, 5.48
T_last,_ h	335, 336	309	25.1, 170
T_max_ h	2.00, 2.03	6.00	1.00, 1.15

^a^Individual patient value(s) are presented when *N* < 3

^b^Only 1 patient had quantifiable C_trough_ concentrations

*AUC*_*last*_ Area under the serum concentration–time profile from time zero to the time of the last quantifiable concentration, *C*_*max*_ Maximum observed serum concentration, *C*_*trough*_ Predose concentration during multiple dosing, *dn* Dose normalized, *n* Number of patients in the treatment group and contributing to the summary statistics, *T*_*last*_ Time of last measurable concentration, *T*_*max*_ Time for C_max_

Following co-administration with utomilumab, mogamulizumab PK was similar across groups, with accumulation observed following multiple-dose administration (Additional file 1 and Additional file 2).

Thirteen of 24 (54.2%) patients that received utomilumab tested positive for treatment-induced ADA and 11 of 24 (45.8%) were positive for NAb. One (4.2%) patient who received mogamulizumab was confirmed positive for treatment-induced ADA; no one in this group tested positive for NAb. The median onset for treatment-induced ADA against utomilumab was 56.01 days (interquartile range [Q1, Q3]: 55.88, 56.13) and the median onset for NAb was 56.13 days (interquartile range [Q1, Q3]: 55.96, 56.97). The median duration of ADA and NAb was 0 and 0 days, respectively (interquartile range [Q1, Q3]: 0, 55.95 for ADA and 0, 62.84 for NAb). There was no substantial impact of ADA/NAb on PK and safety when utomilumab and mogamulizumab were administered in combination.

### Pharmacodynamics

**Fig. 1:**
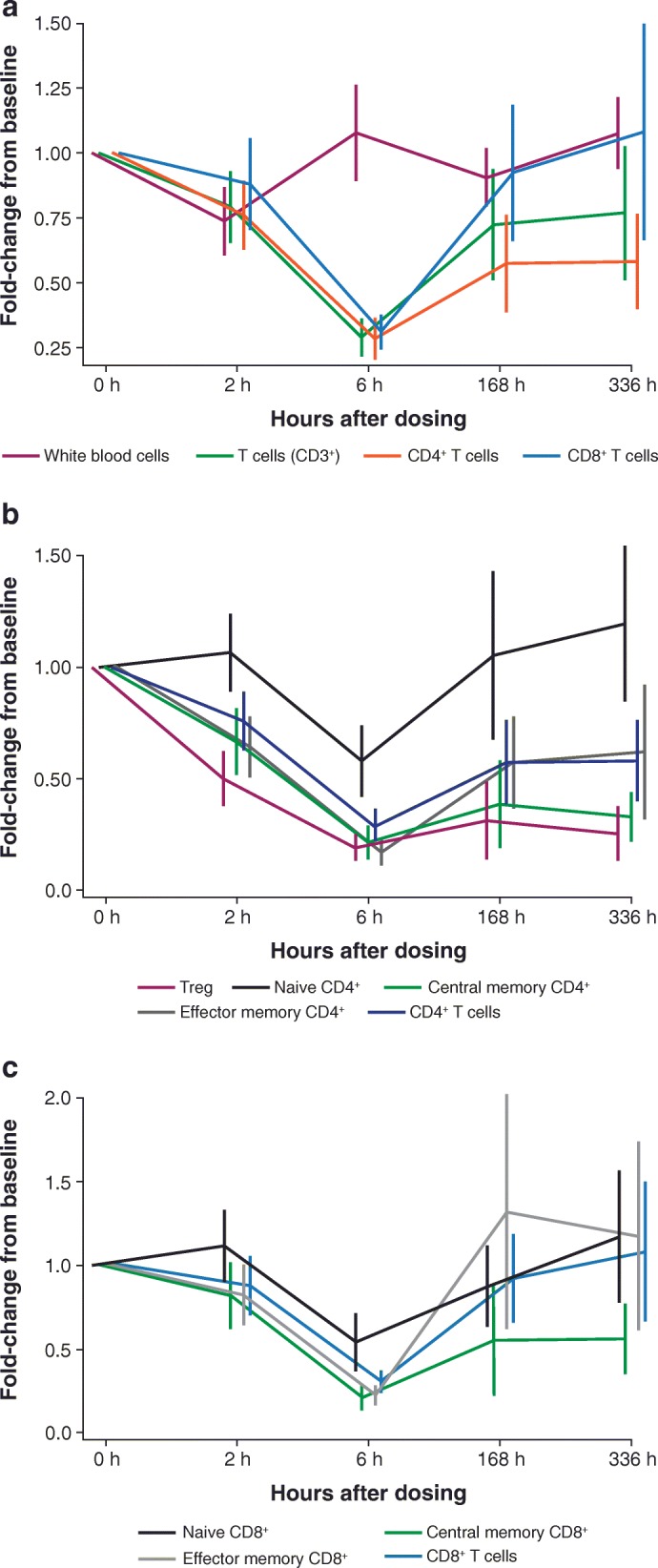
Fold-changes relative to baseline. Fold-changes are shown by lymphocyte populations in peripheral blood following treatment with utomilumab and mogamulizumab. Results were aggregated across all utomilumab doses, as statistically significant differences between utomilumab doses were not observed. **a** Major T-cell subpopulations relative to all white blood cells; **b** Treg and other major CD4^+^ T- cell populations; and (**c**) major CD8^+^ T-cell populations. White blood cells were defined by forward and side light scatter. T cells were defined by co-expression of CD3, CD4, and CD8. Naïve, central memory, and effector memory T-cell subpopulations were defined as CD45RA^+^CCR7^+^, CD45RA^−^CCR7^+^, and CD45RA^−^CCR7^−^, respectively [32]. Tregs were defined as CD3^+^CD4^+^CD25^+^CD127^low/^ −[33]. Treg, regulatory T cell

Patients treated with combination utomilumab/mogamulizumab showed a transient reduction in circulating T cells at 6 h (*p* < 0.0001) (Fig. 1a), possibly due to cytokine-induced adhesion of T cells to endothelial cells [34]. There was not a significant (*p* < 0.05) relationship between combination dose, T-cell reduction, and cytokine levels observed in this study (data on file; Pfizer). Levels of circulating CD8^+^ T cells largely returned to baseline by 7 days (168 h) after start of dosing; however, circulating CD4^+^ T cells did not fully recover by 336 h. Statistically significant (*p* < 0.001) decreases in Tregs (CD3 + CD4 + CD25 + CD127^low/−^) were observed at 6, 168, and 336 h **(**Fig. 1b), as predicted based on data from a phase I study of single-agent mogamulizumab in patients with CCR4^−^ lung and esophageal cancers [25]. Reductions in effector memory (CD45RA^−^CCR7^−^) and central memory (CD45RA^−^CCR7^+^) CD4 T cells were also observed at the same time points (*p* < 0.01). Naïve (CD45RA^+^CCR7^+^) CD4^+^ T cells were less strongly affected. Within the CD8^+^ T-cell compartment, the central memory subpopulation was reduced, relative to baseline, at 6, 168, and 336 h (*p* < 0.005) to a greater degree than either the naïve or effector memory compartments **(**Fig. 1c). These results are largely concordant with earlier findings [25] and consistent with observations that CCR4 is expressed by central memory T cells [35, 36].

**Fig. 2:**
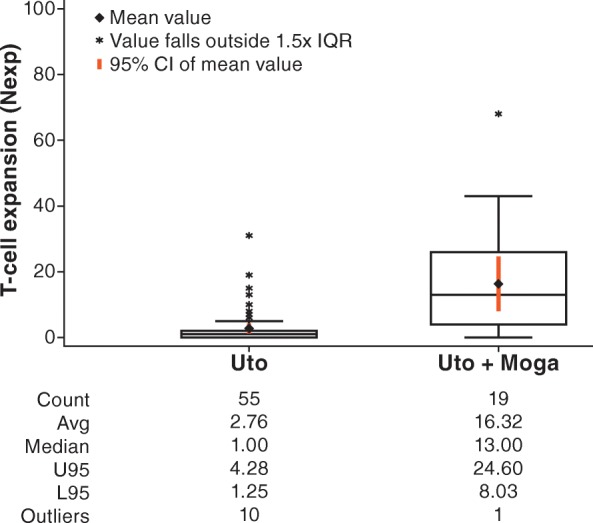
TCR expansion in peripheral blood in a cohort of patients treated for 1 cycle (4 weeks) with utomilumab single-agent [22] vs patients treated with utomilumab/mogamulizumab. Individual TCR sequences were considered to have expanded after treatment if frequencies in on-treatment specimens were greater than normal biologic time-dependent variance as determined by a beta binomial model [28]. Box plot provides median and 25%/75% quartiles with whiskers to the last data point within 1.5× the IQR. CI, confidence interval; IQR, interquartile range; Moga, mogamulizumab; TCR, T-cell receptor; Uto, utomilumab

Longitudinal analysis of TCRβ CDR3 sequences in peripheral blood by immunosequencing has been used to track individual responses to a yellow fever vaccine without prior knowledge of antigen specificity [37]. Immunosequencing was performed on peripheral blood specimens from study patients, and expanded TCRβ CDR3 sequences were identified using a beta binomial model that controls for normal biologic variance over time [28]. Comparison of the number of expanded clones in patients treated with combination utomilumab/mogamulizumab to the number of expanded clones in patients treated with single-agent utomilumab [22] suggests that the addition of mogamulizumab could promote peripheral T-cell expansion (*p* < 0.001), **(**Fig. 2**).**

### Characterization of baseline tumor biopsies

Baseline tumor biopsies from patients with NSCLC (*n* = 1) and SCCHN (*n* = 4) were analyzed for the presence of PD-L1, CD8, FoxP3, and 4-1BB/CD137 **(**Table 3**)**. All biopsies were negative for PD-L1 expression on tumor cells, except for one SCCHN specimen that had 10% PD-L1^+^ tumor cells. The biopsies had low levels of infiltrating CD8^+^ (range, 1–17%) and FoxP3^+^ (range, 1–9%) cells, with CD8/FoxP3 ratios ranging from 1 to 9. Also, 4-1BB/CD137 was observed on small percentages (9, 10, and 14%) of cells in the IM. The limited number of available specimens precludes generalizations about the cohort.

**Table 3:** Immunohistochemistry analysis of whole tumor and IM of pretreatment tumor biopsies

Cancer Type	BOR	%PD-L1^+^	%CD8^+^ ALL^a^	%FoxP3^+^ ALL^a^	CD8/FoxP3 ALL^a^	%CB8^+^ IM^b^	%FoxP3^+^ IM^b^	%CD137^+^ IM^b^	CD8/FoxP3 IM^b^
NSCLC	PR	0.00	1.70	1.72	0.99	9.96	1.45	14.21	6.87
SCCHN	PD	0.00	6.89	5.38	1.28	13.28	6.48	8.59	2.05
SCCHN	SD	0.00	17.16	1.74	9.86	NE	NE	NE	NE
SCCHN	SD	10.00	13.69	4.98	2.75	NE	NE	NE	NE
SCCHN	SD	0.00	12.23	9.43	1.3	14.68	10.05	10.37	1.46

Marker-positive cells are reported as a percent of evaluated cells

^a^ALL: The region encompassing the tumor and extending up to the leading edge, but not outside the tumor-normal interface

^b^IM: The region extending from 500 μm outside the leading edge of the tumor to 500 μm inside

*BOR* Best overall response, *NE* Not evaluable, *NSCLC* Non–small-cell lung cancer, *PD* Progressive disease, *PD-L1* Programmed cell death-ligand 1, *PR* Partial response, *SCCHN* Squamous cell cancer of head and neck, *SD* Stable disease

### Efficacy

**Fig. 3:**
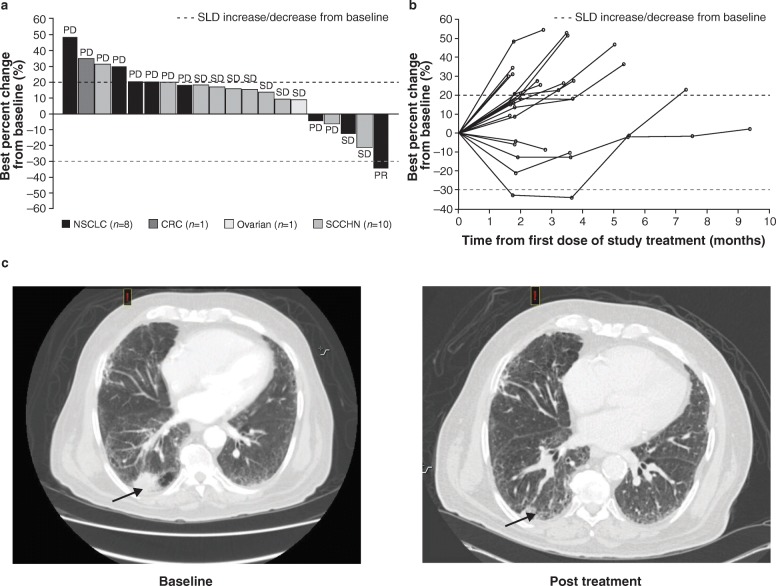
Antitumor efficacy. A BOR of partial response was observed in 1 patient with PD-1–refractory squamous NSCLC. **a** Waterfall plot of best percent change from baseline in the SLD for target lesions, full analysis set, with BOR indicated for each patient. **b** Spider plot of percent change from baseline in SLD for target lesions over time, full analysis set. **c** Baseline and post-treatment scan images. The baseline scan was taken ~ 4 weeks prior to treatment initiation. The post-treatment scan was taken at ~ 7 weeks after treatment initiation. Arrows point to tumor location in right lower lobe of the lung. CRC, colorectal cancer; NSCLC, non–small-cell lung cancer; PD-1, programmed cell death 1; SCCHN, squamous cell cancer of the head and neck; SLD, sum of the longest diameter

The ORR was 4.2% (95% CI: 0.1–21.1%). Best percent change from baseline in sum of longest diameters (SLD) for target lesions is shown in the waterfall plot **(**Fig. 3a). The spider plot **(**Fig. 3b) shows percentage change from baseline in sum of SLD for target lesions over time. One patient in the utomilumab 100 mg/mogamulizumab 1 mg/kg group with PD-1 refractory squamous NSCLC achieved PR, which occurred at the first tumor assessment with a duration of response of approximately 2 months.

The patient with PR had previously received carboplatin and paclitaxel as first-line therapy (BOR of PR) and nivolumab as second-line therapy (BOR of progressive disease), and prior brain radiotherapy. This patient tested positive for both ADA and NAb against utomiliumab. The tumor was negative for PD-L1, with low levels of infiltrating T lymphocytes while demonstrating relatively higher numbers of 4-1BB/CD137^+^ cells and an elevated CD8/FoxP3 ratio **(**Table 3**)**. No patients achieved a BOR of CR. Nine patients had BOR of SD, 10 patients had BOR of progressive disease, and 4 were not evaluable. Representative baseline and post-treatment scans highlighting tumor shrinkage in the patient achieving PR are shown in Fig. 3c.

## Discussion

In this phase I study of the combination of utomilumab with mogamulizumab in patients with advanced solid tumors, the MTD for utomilumab was determined to be at least 2.4 mg/kg, and utomilumab doses up to 5 mg/kg combined with mogamulizumab 1 mg/kg were well tolerated. None of the patients experienced a DLT with any dose combination. Utomilumab systemic exposure appeared to increase with each dose escalation, but the relationship between PK and dose could not be fully evaluated owing to low patient numbers. Following co-administration with utomilumab, mogamulizumab PK was similar across dose groups, with accumulation observed following multiple-dose administration. There was no substantial impact of ADA/NAb on PK and safety.

The peripheral blood biomarker analyses performed in this study indicated that Tregs and at least some central memory T cells were depleted, as was observed by Kurose et al. in patients treated with single-agent mogamulizumab [25]. Expansion of TCRβ CDR3 regions in the combination cohort is consistent with the hypothesis that mogamulizumab-mediated depletion of Tregs and other CCR4^+^ cells can promote peripheral T-cell expansion, although the durability of such expansion in conjunction with potential central memory depletion cannot be assessed.

Pretreatment tumor biopsy results were only available for 5 of the 24 enrolled patients. Four tumor biopsies were PD-L1-negative, including the biopsy from the patient with NSCLC who achieved PR. One patient with SCCHN who achieved BOR of SD had a biopsy with a 10% PD-L1 tumor proportion score. It is possible that many, if not most, of the enrolled patients had tumors with minimal antitumor immune activity. The efficacy of Treg depletion in such tumors is likely contingent on the relationship between Tregs and that phenotype: if Tregs are the primary causal agent, then removing them should increase immune activity, but not if immune activity is reduced for other reasons. The two hypotheses cannot be differentiated in this study. The patient with NSCLC who achieved PR had the highest CD8/FoxP3 ratio and proportion of 4-1BB/CD137^+^ cells in the IM, coupled with the lowest CD8/FoxP3 ratio throughout the tumor itself. This phenotype may suggest the existence of a utomilumab-responsive tumor-infiltrating lymphocyte population in the IM that is being quenched by Tregs closer to the tumor center. A larger, prospectively designed study would be required for further definition of this phenotype and estimation of its prevalence.

This study was designed to test the hypothesis that depletion of CCR4^+^ Tregs would enhance the effect of anti-tumor T cells expanded in response to a 4-1BB agonist. While the observed depletion of Tregs coupled with TCR expansion in the peripheral blood is consistent with this hypothesis, it is possible that other effects of CCR4 depletion may affect clinical outcome. For instance, depletion of CCR4^+^ T cell types, such as memory, Th1, Th2, and resident memory T cells [38–40] could impact the anti-tumor response elicited by combination therapy. It has been reported that CCR4 is required for optimal T cell-mediated protection from influenza in mice [41], and surface CCR4 expression has been observed on lymphocytes isolated from lung and bronchoalveolar lavage fluid [42]. The transience of the PR seen in a PD-1 refractory squamous NSCLC patient may be consistent with attenuation of anti-tumor activity mediated by CCR4-expressing T cells. It is also possible that depletion of CCR4-expressing T cells leaves other tumor-infiltrating Tregs unaffected, such as the CCR8-expressing Tregs that have been noted in multiple tumor types [43, 44].

## Conclusion

The combination of utomilumab plus mogamulizumab was well tolerated in patients with advanced solid tumors, with a PR achieved by 1 NSCLC patient. The results of the translational analyses are consistent with the hypothesized mechanism of action. Clinical benefit from this combination may be meaningful for patients in whom CCR4+ Tregs have induced a dormant CD8^low^/PD-L1^low^ phenotype that may be unresponsive to anti-PD-1/PD-L1 therapy.

## Supplementary information

**Additional file 1.** Descriptive summary of serum mogamulizumab pharmacokinetic parameter values for Cycle 1, single dose. Table of PK values after one dose.

**Additional file 2.** Descriptive summary of serum mogamulizumab pharmacokinetic parameter values for Cycle 5 (biweekly dosing). Table of PK values after multiple dosing.

## Data Availability

Upon request, and subject to certain criteria, conditions and exceptions (see https://www.pfizer.com/science/clinical-trials/trial-data-and-results for more information), Pfizer will provide access to individual de-identified participant data from Pfizer-sponsored global interventional clinical studies conducted for medicines, vaccines and medical devices (1) for indications that have been approved in the US and/or EU or (2) in programs that have been terminated (i.e., development for all indications has been discontinued). Pfizer will also consider requests for the protocol, data dictionary, and statistical analysis plan. Data may be requested from Pfizer trials 24 months after study completion. The de-identified participant data will be made available to researchers whose proposals meet the research criteria and other conditions, and for which an exception does not apply, via a secure portal. To gain access, data requestors must enter into a data access agreement with Pfizer.

## Abbreviations

ADA: Antidrug antibody
AE: Adverse event
ATL: Adult-cell leukemia-lymphoma
BOR: Best overall response
CCR4: C-C chemokine receptor 4
CI: Confidence interval
CR: Complete response
CRC: Colorectal cancer
DLT: Dose-limiting toxicity
ECL: Electrochemiluminescence
EOT: End of treatment
IM: Invasive margin
IQR: Interquartile range
KHK: Kyowa Hakko Kirin
mAB: Monoclonal antibody
Moga: Mogamulizumab
MTD: Maximum tolerated dose
NAb: Neutralizing antibody
NSCLC: Non–small-cell lung cancer
ORR: Objective response rate
PD: Progressive disease
PD-1: Programmed cell death 1
PD-L1: Programmed cell death-ligand 1
PK: Pharmacokinetics
PR: Partial response
r/r: Relapse/refractory
SCCHN: Squamous cell carcinoma of head and neck
SD: Stable disease
SLD: Sum of the longest diameter
TCR: T-cell receptor
TITE-CRM: Time-to-event continual reassessment method
Treg: Regulated T cell
Uto: Utomilumab
