# Supplementary material for: A phase Ib study of utomilumab (PF-05082566) in combination with mogamulizumab in patients with advanced solid tumors
Source: J Immunother Cancer. 2019 Dec 4;7:342. doi: 10.1186/s40425-019-0815-6 (PMC6894203; doi:10.1186/s40425-019-0815-6)
Supplement: Supplementary file 1 — Additional file 1. Descriptive summary of serum mogamulizumab pharmacokinetic parameter values for Cycle 1, single dose. Table of PK values after one dose. [file 40425_2019_815_MOESM1_ESM.pdf]

Additional file 1. Descriptive summary of serum mogamulizumab pharmacokinetic parameter values for Cycle 1, single dose.

	**Mogamulizumab 1 mg/kg + Utomilumab, by Dose[a]**			
**Parameter, Units**	**1.2 mg/kg**	**100 mg**	**2.4 mg/kg**	**5 mg/kg**
N	11	5	4	3
AUC_{last}, µg·h/mL	2001 (24)	2182 (36)	1382 (133)	2126 (24)
AUC_{168}, µg·h/mL	1945 (25)	2124 (33)	2353 (14)[b]	2123 (24)
C_{max}, µg/mL	18.78 (19)	19.73 (32)	19.77 (16)	19.82 (19)
T_{last}, h	166 (164–285)	166 (163–214)	143 (22.4–194)	166 (166–171)
T_{max} h	1.28 (1.00–5.50)	1.30 (1.10–2.25)	1.24 (1.15–4.58)	1.03 (0.92–1.18)

[a] Geometric mean (geometric %CV) for all except: median (range) for T_{max} and T_{last}.

[b] Only three patients contributed to the summary statistics for AUC_{168}.

%CV, percent of coefficient of variation; AUC_{168}, area under the serum concentration–time profile from time zero to 168 hours post dose; AUC_{last}, area under the serum concentration–time profile from time zero to the time of the last quantifiable concentration; C_{max}, maximum observed serum concentration; *N*, number of patients in the treatment group and contributing to the summary statistics; T_{last}, time of last measurable concentration; T_{max}, time for C_{max}
