# Supplementary material for: A phase Ib study of utomilumab (PF-05082566) in combination with mogamulizumab in patients with advanced solid tumors
Source: J Immunother Cancer. 2019 Dec 4;7:342. doi: 10.1186/s40425-019-0815-6 (PMC6894203; doi:10.1186/s40425-019-0815-6)
Supplement: Supplementary file 2 — Additional file 2. Descriptive summary of serum mogamulizumab pharmacokinetic parameter values for Cycle 5 (biweekly dosing). Table of PK values after multiple dosing. [file 40425_2019_815_MOESM2_ESM.pdf]

Additional file 2 Descriptive summary of serum mogamulizumab pharmacokinetic parameter values for Cycle 5 (biweekly dosing).

	Mogamulizumab 1 mg/kg + Utomilumab, by Dose[a]		
**Parameter, Units**	**1.2 mg/kg**	**100 mg**	**5 mg/kg**
N, *n*	2, 2	1, 1	2, 1
AUC_{last}, µg·h/mL	6390, 7350	5870	1170, 4560
AUC_{τ}, µg·h/mL	6400, 7370	6160	6950[b]
C_{max}, µg/mL	28.7, 33.6	31.9	28.3, 37.6
CL, mL/h/kg	0.136, 0.156	0.162	0.144[b]
C_{trough}, µg/mL	15.4, 15.5	9.04	10.4, 24.6
T_{last}, h	334, 335	308	45.2, 168
T_{max}, h	4.52, 22.4	1.05	1.02, 1.07

[a] Individual patient value(s) are presented when N<3.
[b] Only one patient in the treatment had reportable AUC_{τ} and CL values.
AUC_{last}, area under the serum concentration–time profile from time zero to the time of the last quantifiable concentration; AUC_{τ}, area under the serum concentration–time profile from time zero to time tau (τ), the dosing interval; CL, clearance; C_{max}, maximum observed serum concentration; C_{trough}, pre-dose concentration during multiple dosing; N, number of patients in the treatment group and contributing to the summary statistics; n, number of patients with reportable CL values; T_{last}, time of last measurable concentration; T_{max}, time for C_{max}.
